# Correction to “Prevalence, Antimicrobial Resistance, and Staphylococcal Toxin Genes of *Bla*_TEM‐1a_‐producing *Staphylococcus aureus* Isolated From Animals in Chongqing, China”

**DOI:** 10.1002/vms3.70653

**Published:** 2025-10-21

Dong, Q., Wang, Q., Zhang, Y., Chen, Y., Wang, H., & Ding, H. (2023). Prevalence, antimicrobial resistance, and staphylococcal toxin genes of bla_TEM‐1a_‐producing Staphylococcus aureus isolated from animals in Chongqing, China. *Veterinary Medicine and Science*,9,513‐522. https://doi.org/10.1002/vms3.1028

Some of the results in this article are translated from a prior publication published in *Acta Veterinaria et Zootechnica Sinica* (Chen et al. 2018 [10.11843/j.issn.0366‐6964.2018.05.023]). Guidance from the Committee on Publication Ethics notes that original sources should always be acknowledged for translations (Publishing translations of previously published articles and resources | COPE: Committee on Publication Ethics). The original article was published under a CC BY‐NC‐ND license, and the license terms stipulate that when redistributing the material, users must “give appropriate credit, provide a link to the license, and indicate if changes were made” (Deed—Attribution‐NonCommercial‐NoDerivatives 4.0 International—Creative Commons).

We apologize for this error.

